# QuickRNASeq lifts large-scale RNA-seq data analyses to the next level of automation and interactive visualization

**DOI:** 10.1186/s12864-015-2356-9

**Published:** 2016-01-08

**Authors:** Shanrong Zhao, Li Xi, Jie Quan, Hualin Xi, Ying Zhang, David von Schack, Michael Vincent, Baohong Zhang

**Affiliations:** PharmaTherapeutics Clinical R&D, Pfizer Worldwide Research and Development, Cambridge, MA 02139 USA; Computational Sciences Center of Emphasis, Pfizer Worldwide Research and Development, Cambridge, MA 02139 USA

**Keywords:** RNA-seq, Pipeline, Workflow, Automation, Visualization, Batch processing, High-performance computing, Large-scale data analysis, D3, jQuery

## Abstract

**Background:**

RNA sequencing (RNA-seq), a next-generation sequencing technique for transcriptome profiling, is being increasingly used, in part driven by the decreasing cost of sequencing. Nevertheless, the analysis of the massive amounts of data generated by large-scale RNA-seq remains a challenge. Multiple algorithms pertinent to basic analyses have been developed, and there is an increasing need to automate the use of these tools so as to obtain results in an efficient and user friendly manner. Increased automation and improved visualization of the results will help make the results and findings of the analyses readily available to experimental scientists.

**Results:**

By combing the best open source tools developed for RNA-seq data analyses and the most advanced web 2.0 technologies, we have implemented QuickRNASeq, a pipeline for large-scale RNA-seq data analyses and visualization. The QuickRNASeq workflow consists of three main steps. In Step #1, each individual sample is processed, including mapping RNA-seq reads to a reference genome, counting the numbers of mapped reads, quality control of the aligned reads, and SNP (single nucleotide polymorphism) calling. Step #1 is computationally intensive, and can be processed in parallel. In Step #2, the results from individual samples are merged, and an integrated and interactive project report is generated. All analyses results in the report are accessible via a single HTML entry webpage. Step #3 is the data interpretation and presentation step. The rich visualization features implemented here allow end users to interactively explore the results of RNA-seq data analyses, and to gain more insights into RNA-seq datasets. In addition, we used a real world dataset to demonstrate the simplicity and efficiency of QuickRNASeq in RNA-seq data analyses and interactive visualizations. The seamless integration of automated capabilites with interactive visualizations in QuickRNASeq is not available in other published RNA-seq pipelines.

**Conclusion:**

The high degree of automation and interactivity in QuickRNASeq leads to a substantial reduction in the time and effort required prior to further downstream analyses and interpretation of the analyses findings. QuickRNASeq advances primary RNA-seq data analyses to the next level of automation, and is mature for public release and adoption.

## Background

RNA sequencing (RNA-seq) has emerged as a powerful technology in transcriptome profiling [1–3]. Our previous side-by-side comparison between RNA-seq and microarray in investigating T cell activation demonstrated that RNA-seq analysis has many advantages over microarray analysis [4]. In contrast to hybridization-based microarray analyses, RNA-seq has the extra benefits of obtaining transcription start and stop sites, alternative spliced isoforms, and genetic variants in addition to gene expression levels. One apparent shortcoming of early non-stranded (standard) RNA-seq protocols is that a sequence read loses the strand origin information, thus making it difficult to determine accurately the expression levels of overlapping genes transcribed from opposite strands. A comparison of stranded with non-stranded RNA-seq led us to conclude that stranded RNA-seq provides a more accurate estimation of gene expression levels than non-stranded RNA-seq [5].

Short reads generated by RNA-seq experiments must first be aligned, or mapped, to a reference genome or transcriptome assembly. The general objective of mapping or aligning a collection of sequence reads to a reference is to discover the true location (origin) of each read with respect to that reference. Although a large number of read mapping algorithms have been developed in recent years [6–10], the accurate alignment of RNA-seq reads is still a challenge. Indeed, some features of a reference genome such as repetitive regions, assembly errors, and assembly gaps render this objective impossible for a subset of reads. Furthermore, because RNA-seq libraries are constructed from transcribed RNA, intronic sequences are not present in exon-exon spanning reads. Therefore, when aligning the sequences to a reference genome, reads that span exon-exon junctions have to be split across potentially thousands of bases of intronic sequence. Many of the RNA-seq alignment tools, including STAR [11], GSNAP [12], MapSplice [13], and TopHat [14], use reference transcriptomes to inform the alignment of junction reads. The benefits of using a reference transcriptome to map RNA-seq reads have been demonstrated clearly in our previous RNA-seq analyses [15, 16].

The second important step in most RNA-seq analyses is gene or isoform quantification. A common method to estimate gene or transcript abundance from RNA-seq data is to count the number of reads that map uniquely to each gene or transcript. RPKM (reads per kilobase per million reads) is widely used to represent the relative abundance of mRNAs for a gene or transcript. A number of algorithms have been developed to infer gene and isoform abundance [17, 18], including RSEM [19, 20], Cufflinks [21], IsoEM [22], featureCounts [23], and HTSeq [24]. A gene can be expressed in one or more transcript isoforms; accordingly, its expression level should be represented as the sum of its isoforms. However, estimating the expression of individual isoforms is intrinsically more difficult because different isoforms of a gene typically have a high proportion of genomic overlap. Accordingly, a simpler union exon-based approach has been proposed, in which all overlapping exons of the same gene are first merged into union exons, and the total length of the union exons is taken to represent the gene length. We carried out a side-by-side comparison between union exon-based approach and transcript-based method in RNA-seq gene quantification [25], and found that gene expressions were significantly underestimated when the union exon-based approach was used. Therefore, we strongly discourage using the union exon-based approach in gene quantification despite its simplicity.

Although the time and cost for generating RNA-seq data are decreasing, the analysis of massive amounts of RNA-seq data still remains challenging. Numerous software packages and algorithms for basic data quality control (QC) and analyses have been developed, which has led to the need to apply these tools efficiently to obtain results within a reasonable timeframe, especially for large datasets. Based on our own experience with in-house analyses of multiple RNA-seq datasets of varying size using open source tools, the main challenges, gaps, and bottlenecks for large-scale RNA-seq data analyses can be summarized as follows:

1. Selecting appropriate software packages and setting software-specific parameters. Making the right or best choice can be difficult because many similar tools are available. Setting software parameters is even harder if not impossible, because it often requires both an in-depth understanding of the algorithms and sufficient hands-on experience, which disadvantages researchers new to this field.
2. Writing scripts to make different components work seamlessly in a pipeline. A variety of algorithms have been designed to perform different tasks, but they have been developed (and/or maintained) independently by different research groups and often use different programming languages. Moreover, those algorithms do not understand each other well, and the output(s) from one algorithm often cannot be used as input(s) for another algorithm. As a result, additional bridging scripts are necessary, which ideally requires a data analyst who is familiar with a number of programming languages, including Shell script, Perl, Python, Java, C/C++, and R.
3. Integrating and summarizing analyses results from individual samples. In general, most algorithms are implemented to process an individual sample. Consequently, the results of primary RNA-seq data analyses have to be further processed, integrated, and summarized for reporting, presentation, and downstream analysis. Usually, data integration and summarization are tedious and not easy to execute efficiently.
4. Identifying RNA-seq sample outliers. It is not uncommon that some samples have low quality and often substitute samples are not available, especially for RNA-seq of clinical specimen. RNA-seq is a complicated multistep process that involves sample collection/stabilization, RNA extraction, fragmentation, cDNA synthesis, adapter ligation, amplification, purification, and sequencing. Any mistake in this complex sequence of protocols can result in biased or even unusable data. Therefore, it is necessary to establish stringent RNA-seq data quality metrics to identify outliers that should be excluded from further downstream data analysis.
5. Detecting sample swapping and mislabeling. For large-scale RNA-seq studies in which hundreds or even thousands of RNA samples are sequenced and analyzed, it is not unusual that some samples are mishandled and appear to be swapped or sequenced more than once. Such errors can become a serious problem for downstream data analyses and interpretation of results, especially for longitudinal sample analyses. It is difficult to identify such mistakes based only on RNA-seq QC metrics and/or gene expression profiles. To confirm whether samples are from the same subject, it is more reliable to compare genetic markers among samples, such as single nucleotide polymorphisms (SNPs).
6. Sharing the results of RNA-seq data analyses with experimental scientists. Nearly all RNA-seq data analyses are performed using Linux clusters or workstations; however, analyses results in Linux are often inaccessible to most experimental scientists. RNA-seq data analyses typically generate a large number of files and large amounts of data that are difficult to comprehend or digest directly by experimental scientists. Therefore, easily accessible interfaces are needed that not only provide a quick and easy way for non-expert users to obtain high-level visualizations of the main RNA-seq analyses outputs (e.g., QC results), but also allow them to drill down further or export the results into additional analysis applications of their choice. To the best of our knowledge, very few RNA-seq related open source packages provide all these options.

To address these challenges, we have implemented a new pipeline named QuickRNASeq to advance the automation and visualization of RNA-seq data analyses results, and have constantly improved and refined its implementation since its inception. QuickRNASeq significantly reduces data analysts’ hands-on time, which results in a substantial decrease in the time and effort needed for the primary analyses of RNA-seq data before proceeding to further downstream analysis and interpretation. Additionally, QuickRNASeq provides a dynamic data sharing and interactive visualization environment for end users. All the results are accessible from a web browser without the need to set up a web server and database. The rich visualization features implemented in QuickRNASeq enable non-expert end users to interact easily with the RNA-seq data analyses results, and to drill down into specific aspects to gain insights into often complex datasets simply through a point-and-click approach.

## Implementation

**Fig. 1:**
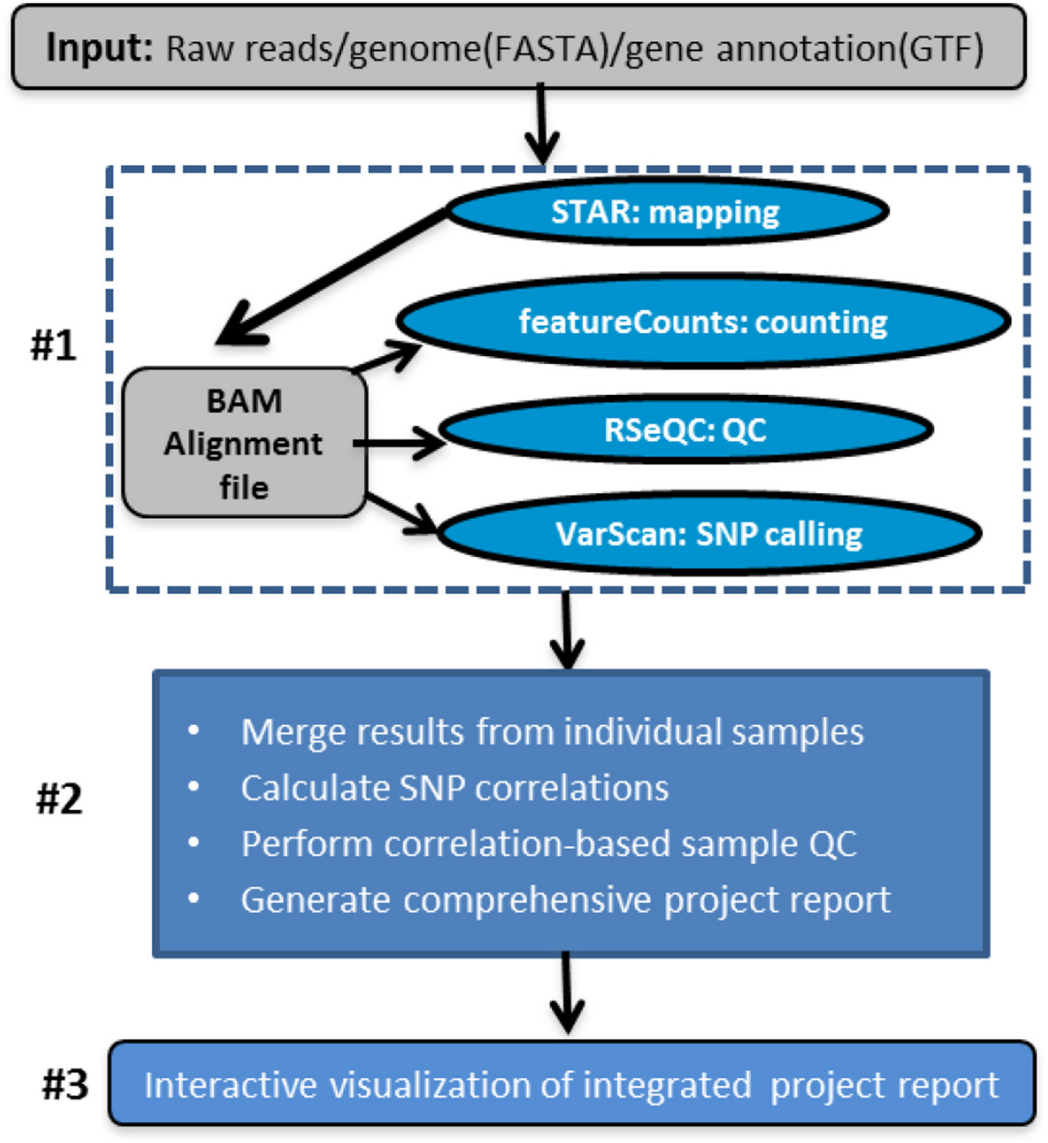
Overview of the QuickRNASeq pipeline. Step #1 is computationally intensive, and processes individual samples independently. Step #2 integrates RNA-seq data analysis results from the individual samples in Step #1 and generates a comprehensive project report. Step #3 offers interactive navigation and visualization of RNA-seq data analyses results

QuickRNASeq is designed for simplicity and visual interactivity. A few important principles dictate its implementation. First, all components of the pipeline are freely available in the public domain. Second, it is easy to deploy and use. Third, all analyses results including RNA-seq QC metrics, sample correlations, and gene quantifications are accessible via a web browser and can be further explored interactively. An overview of QuickRNASeq (Fig. 1) illustrates its three main steps. Step #1 performs RNA-seq read mapping, counting, aligned read QC, and SNP calling. Step #1 processes each sample completely independently of each other, and is computationally intensive. Therefore, all samples can be processed in parallel in a high performance computing cluster (HPC), or in a serial fashion on a standalone workstation. Step #2 merges the results from the individual sample and generates an integrated and interactive project report for data interpretation in Step #3.

### Input files

In addition to raw sequence reads in FASTQ format, the only other required inputs are a reference genome file in FASTA format and a corresponding gene annotation file in GTF (gene transfer format). QuickRNASeq can be applied to any species as long as its genome and gene annotations are available; for example, human, mouse, rat, and cynomolgus or rhesus monkeys. A gene annotation file can exist in many formats, but GTF has become the de facto standard; however, not all tools accept gene annotation files in GTF format as input. For example, RSeQC (RNA-seq quality control package) [26] accepts gene annotation only in BED (browser extensible display) format, though the majority of gene annotations in the public domain are not available in BED format. To ensure that the exact same annotations are used by the different components in QuickRNASeq, we wrote Perl scripts to convert gene annotation files from GTF to BED format. This avoids any discrepancy or inconsistency among gene annotations that are available in different formats.

### Step #1: single sample processing

This step consists mainly of read mapping, counting, aligned read QC, and SNP calling, and the corresponding algorithms used to perform these tasks are STAR [11, 27], featureCounts [23], RSeQC [26], and VarScan [28] respectively. STAR aligns spliced sequences of any length with moderate error rates, provides scalability for emerging sequencing technologies, and generates output files ready for transcript/gene expression quantification [27]. The algorithms featureCounts [23] and HTSeq [24] are comparable in terms of counting results, but featureCounts is considerably faster than HTSeq by an order of magnitude for gene-level summarization and requires far less computer memory. Read mapping and counting typically are very time consuming, and we chose STAR and featureCounts in QuickRNASeq mainly because of their high speed and accuracy.

The RSeQC [26] package provides a number of modules that can comprehensively inspect sequence quality, nucleotide composition bias, PCR bias, GC bias, mapped reads distribution, coverage uniformity, and strand specificity. All such QC metrics are valuable for outlier detection. VarScan [28] is a platform-independent software tool that can detect variants in RNA-seq data. It employs a robust heuristic/statistic approach to call variants that meet desired thresholds for read depth, base quality, variant allele frequency, and statistical significance. To verify samples from the same subject, it is unnecessary to call SNPs across all chromosomes. In practice, it is sufficient to use only SNPs from the chromosome that contains the major histocompatibility complex (MHC) genes. For human, mouse, and rat, these are chromosomes 6, 17, and 20, respectively. As mentioned earlier, numerous software packages that can perform similar tasks are freely available; however, we found that the combination of STAR, featureCounts, RSeQC, and VarScan represents one of the best toolsets.

Computational algorithms for RNA-seq analyses are continuously being improved, including STAR, featureCounts, RSeQC, and VarScan. Therefore, we designed our pipeline to be independent of its underlying software version and ensured that it can handle RNA-seq samples from a variety of species. To decouple the dependence of QuickRNASeq pipeline upon underlying computational algorithms and species, we introduced a plain text configuration file that can store project, species, and software-specific parameters. This configuration file also improves the reproducibility of RNA-seq data analyses and simplifies the command lines in QuickRNASeq. For the convenience of QuickRNASeq users, a configuration file template has been provided for easy customization.

### Step #2: data integration, QC, and summary

Step #2 aims mainly to merge results generated in Step #1 for each individual RNA-seq sample. Additionally, it runs many across-sample calculations, such as correlation-based QC and a SNP correlation matrix. As shown in Fig. 1, the second step performs the following tasks:

1. Merge mapping, counting summaries, and RSeQC metrics from individual samples.
2. Generate a read counting table ready for downstream analysis of all annotated genes.
3. Calculate a SNP (and gene expression) correlation matrix among samples.
4. Perform correlation-based sample QC, calculation of MADScore (median absolute deviation score), and data normalization.
5. Produce RNA-seq metrics and correlation plots ready for PowerPoint presentations.
6. Generate a comprehensive HTML QC report for individual sample.
7. Produce a dynamic and integrated QC metrics plot for individual samples.
8. Generate a master HTML entry webpage for data analyses results.

Each individual task listed above is performed by a corresponding Bash, Perl, or R script, and a master script coordinates the execution of all these tasks. The main scripts and their functions are listed in Table 1. As shown in Table 1, the primary RNA-seq data analyses can be performed by as few as two shell command lines (*star-fc-qc.sh* and *star-fc-qc.summary.sh*). All the plots generated in Step #2 are ready for presentations, and the gene counting table can feed downstream differential analysis algorithms. The highly automated features in Step #2 make QuickRNASeq an efficient tool for typical standard RNA-seq analyses, and our pipeline substantially reduces the hands-on time (not the computational time) that data analysts have to spend on primary RNA-seq data analyses.

**Table 1:** Description of main scripts in the QuickRNASeq package

Script	Function
star-fc-qc.sh	Master script for Step #1 in Fig. 1
star-fc-qc.ws.sh	Same as star-fc-qc.sh, but implemented for a standalone workstation
star-fc-qc.summary.sh	Master script for Step #2 in Fig. 1
get-star-summary.pl	Merge STAR mapping summary
get-fc-summary.pl	Merge featureCounts counting summary
get-read-dist.pl	Merge read distribution from RSeQC
get-snp-corr.pl	Calculate all-against-all pairwise SNP correlations
get-expr-table.R	Merge counts table from individual samples
get-expr-qc.R	Perform correlation-based QC, and calculate normalization factor
plot-rnaseq-metrics.R	Plot the summaries for read mapping, counting, or read distribution
plot-corr-matrix.R	Plot a correlations matrix
plot-expr-count.R	Plot the number of genes with varying RPKM cut-offs
RSeQC-html.pl	Generate a HTML QC report for individual sample
make_HTMLs.sh	Generate a comprehensive, integrated, and interactive project report
gtf2annot.pl	Utility to extract gene annotation from a GTF file
gtf2bed.pl	Utility to convert a gene annotation from GTF to BED format
star-fc-qc.config.template	Template configuration file for customization

We implemented a correlation-based QC to detect potential outliers in the RNA-seq data by calculating a MADScore for each sample. In general, an outlier appears to deviate markedly from other samples in a RNA-seq study, and thus its correlation with other samples will be relatively low. The MADScore is calculated as follows. For each sample, calculate the correlation difference, which is simply the difference between the average of all the pairwise correlations that involve the sample and the average of all the pairwise correlations that do not involve the sample. If a sample is an outlier, then the difference will be negative. Accordingly, there will be a vector of values (one for each sample). Then this vector of difference is converted to MADScores (robust Z-scores) by subtracting the medians and dividing it by median absolute deviations (MAD). A standard MADScore cutoff (e.g., −5) is set to determine the outliers.

### Step #3: interactive data visualization

Primary RNA-seq data analyses results are represented by a standard file folder structure, and an integrated report provides comprehensive QC metrics and a gene expression table. RNA-seq data analyses typically generate a variety of files and large amounts of data, and the master entry webpage generated in Step #2 makes data navigation and visualization more convenient. More importantly, the project report offers interactive visualizations of RNA-seq QC and gene expression levels, and provides analytical tools to gain insights into the data. All required JavaScript libraries have already been packaged into the QuickRNASeq project report; thus, deployment of the data into a web server is optional.

JavaScript has become the hallmark of the web 2.0 technologies because it greatly enhances interactive visualizations. The availability of JavaScript-based open source visualization libraries has fueled the adoption of this technique. We implemented the interactive data visualization in QuickRNASeq using these libraries, including JQuery [29], D3 (Data-Driven Documents) [30], canvasXpress [31], SlickGrid [32], and Nozzle [33]. JQuery [29] makes HTML page traversal, manipulation, event handling, and animation simple. D3 [30] manipulates HTML documents based on input data using HTML5, SVG, and CSS (cascade style sheet). canvasXpress [31] supports a large number of plotting types and offers sample grouping, data transformation, and many other features that are usually only seen in commercial software. SlickGrid [32] is a powerful web-based spreadsheet component that supports searching, sorting, and pagination of tabular datasets, and can be scaled to handle millions of data points. Nozzle [33] is an R package that provides an API (application programming interface) to generate HTML reports with dynamic user interface elements. Nozzle is designed to facilitate summarization and rapid browsing of complex results in data analysis pipelines where multiple analyses are performed frequently on big datasets. By combining these visualization libraries with RNA-seq analyses results, we created multiple dynamic HTML pages to present the RNA-seq QC metrics, and to present gene expression profiles in boxplot and heat map formats dynamically and interactively.

## Results and discussion

### Test run of QuickRNASeq on a publicly available dataset

GENCODE annotation [34, 35] is based on Ensembl [36] but with improved coverage and accuracy, and thus is used by the ENCODE consortium [37] as well as many other projects (e.g., 1000 Genomes [38]) as the reference gene set. Therefore, we chose the GENCODE annotation for our test run. GENCODE Release 19 was downloaded from the GENCODE web site [35]. An analysis of RNA-seq data from 1641 samples across 43 tissues of 175 individuals in the Genotype-Tissue Expression (GTEx) project [39, 40] revealed the landscape of gene expression across tissues, and catalogued thousands of tissue-specific genes. For our test run, we selected 48 GTEx samples from five donors. The sample identifiers, annotations, and RNA-seq mapping summaries for all 48 samples are listed in Table 2. Note that a sequence read can be aligned uniquely to a reference genome, or mapped to multiple locations. Some reads cannot be mapped to the reference genome at all. The percentages of reads that were uniquely mapped, mapped to multiple locations, or unmapped are given in Table 2. The complete report for our test run of the GTEx dataset can be downloaded directly from the QuickRNASeq project home page, and is briefly described below.

**Table 2:** Annotation and mapping summary for the 48 samples used in the QuickRNASeq test run

Sample	Subject	Tissue	Sex	Total_reads	Uniq_Rate^a^	Multi_Rate^b^	Unmap_Rate^c^
SRR607214	GTEX-N7MS	Blood	M	39769361	54.59	23.5	21.91
SRR615261	GTEX-N7MS	Blood Vessel	M	47785162	79.69	2.21	18.1
SRR603068	GTEX-N7MS	Brain	M	53339811	59.45	2.15	38.4
SRR821282	GTEX-N7MS	Esophagus	M	44678159	65.58	2.62	31.8
SRR608096	GTEX-N7MS	Heart	M	58482196	72.91	2.8	24.29
SRR612839	GTEX-N7MS	Muscle	M	52016412	70.81	2.37	26.82
SRR816609	GTEX-N7MS	Pituitary	M	38214685	62.27	2.37	35.36
SRR821518	GTEX-N7MS	Testis	M	61509101	83.31	3.85	12.84
SRR607679	GTEX-N7MS	Thyroid	M	80820067	51.37	2.38	46.25
SRR809283	GTEX-N7MT	Blood	F	48818685	64.62	10.77	24.61
SRR808044	GTEX-N7MT	Blood Vessel	F	44714926	81.42	2.92	15.66
SRR598671	GTEX-N7MT	Brain	F	45163430	70.26	3.12	26.62
SRR598509	GTEX-N7MT	Heart	F	44403911	71.19	4.3	24.51
SRR600784	GTEX-N7MT	Lung	F	28065576	76.74	2.3	20.96
SRR813208	GTEX-N7MT	Pancreas	F	53422565	72.34	4.37	23.29
SRR821573	GTEX-N7MT	Pituitary	F	54452379	85.61	3.52	10.87
SRR810945	GTEX-NFK9	Blood	M	41131423	60.85	18.12	21.03
SRR811819	GTEX-NFK9	Blood Vessel	M	49527122	85.48	2.81	11.71
SRR820689	GTEX-NFK9	Esophagus	M	33541344	81.35	3.4	15.25
SRR602106	GTEX-NFK9	Heart	M	65071994	80.04	4.76	15.2
SRR607166	GTEX-NFK9	Lung	M	58741362	76.22	2.91	20.87
SRR598044	GTEX-NFK9	Muscle	M	58643842	80.85	3.36	15.79
SRR614287	GTEX-NFK9	Nerve	M	47388876	70.58	2.4	27.02
SRR811029	GTEX-NFK9	Pancreas	M	51304957	71.95	7.01	21.04
SRR815280	GTEX-NFK9	Prostate	M	85593813	80.46	4.55	14.99
SRR820839	GTEX-NFK9	Testis	M	51113138	66.02	2.89	31.09
SRR603834	GTEX-NFK9	Thyroid	M	61642193	79.4	3.49	17.11
SRR808836	GTEX-NPJ8	Blood Vessel	M	53974446	80.59	3.31	16.1
SRR598124	GTEX-NPJ8	Brain	M	55608656	65.46	3.1	31.44
SRR817306	GTEX-NPJ8	Esophagus	M	62209065	79.22	3.9	16.88
SRR598148	GTEX-NPJ8	Heart	M	53693956	68.13	3.35	28.52
SRR603750	GTEX-NPJ8	Lung	M	25962857	67.55	3.24	29.21
SRR601695	GTEX-NPJ8	Muscle	M	96240522	43.22	1.77	55.01
SRR615790	GTEX-NPJ8	Nerve	M	61182017	58.84	2.45	38.71
SRR819771	GTEX-NPJ8	Pancreas	M	60265701	80.07	4.82	15.11
SRR807949	GTEX-NPJ8	Pituitary	M	95246707	85.12	3.44	11.44
SRR820234	GTEX-NPJ8	Prostate	M	60423220	79.72	3.97	16.31
SRR810899	GTEX-NPJ8	Testis	M	57950635	81.5	3.71	14.79
SRR602951	GTEX-NPJ8	Thyroid	M	100317976	38.72	2.1	59.18
SRR815494	GTEX-O5YT	Blood	M	61808169	65.24	4.9	29.86
SRR809785	GTEX-O5YT	Blood Vessel	M	60730604	86.73	2.59	10.68
SRR814003	GTEX-O5YT	Esophagus	M	64985455	85.69	3.07	11.24
SRR820316	GTEX-O5YT	Heart	M	66455677	81.96	2.79	15.25
SRR821525	GTEX-O5YT	Lung	M	56250586	78.65	2.75	18.6
SRR815044	GTEX-O5YT	Muscle	M	65449073	84.77	2.96	12.27
SRR812080	GTEX-O5YT	Nerve	M	58246823	86.85	3.1	10.05
SRR810761	GTEX-O5YT	Pancreas	M	64065959	73.8	5.49	20.71
SRR818850	GTEX-O5YT	Testis	M	64388347	84.18	3.52	12.3

The samples are from the Genotype-Tissue Expression (GTEx) project [39, 40]

^a^Uniq_Rate, percentage of reads that were uniquely mapped. ^b^Multi_Rate, percentage of reads mapped to multiple locations. ^c^Unmap_Rate, percentage of unmapped reads

#### All analyses results accessible from a single entry webpage

**Fig. 2:**
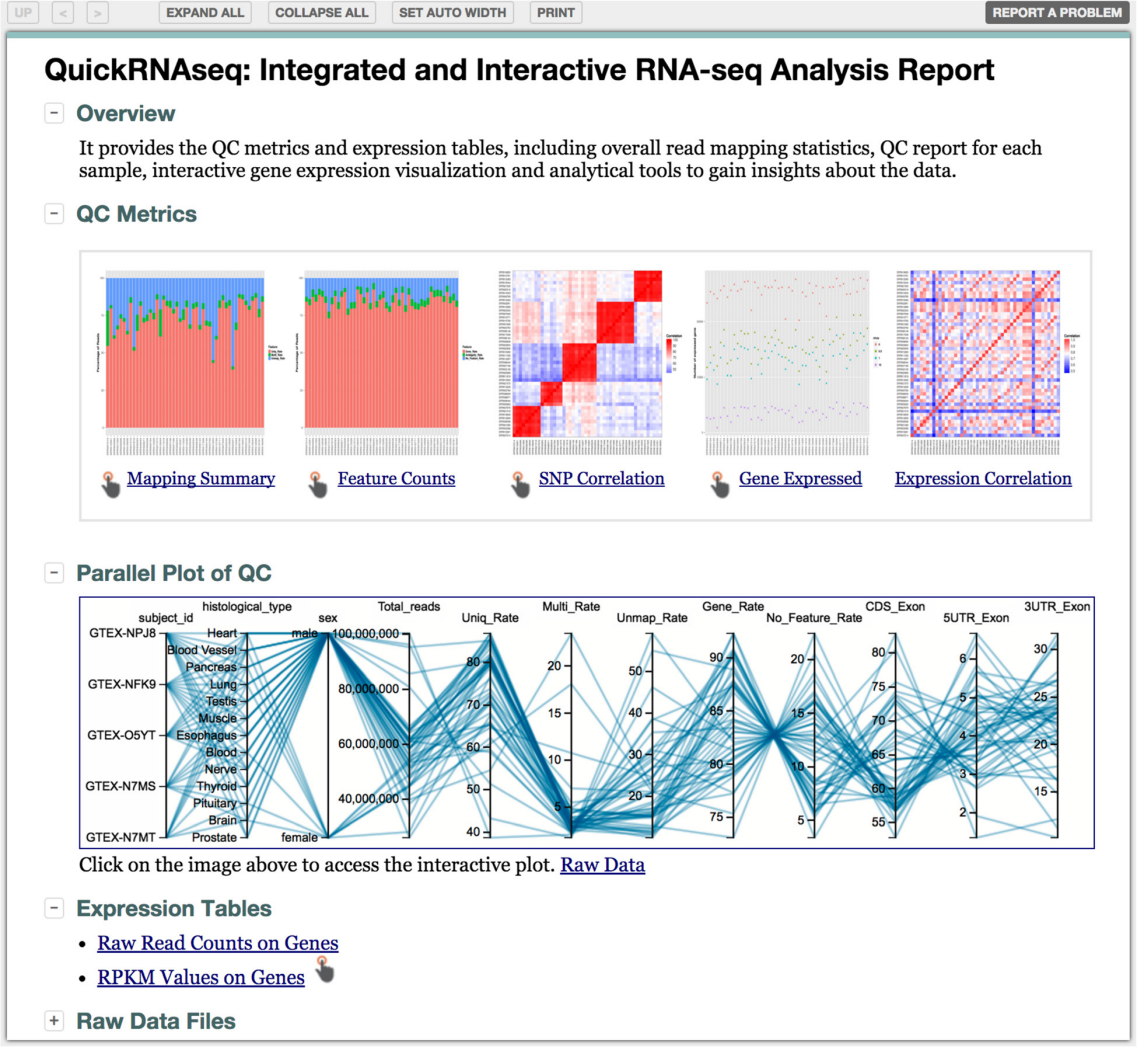
Representative entry webpage for a QuickRNAseq project report. The page layout and printable version of the page can be controlled by the top icons. The QC Metrics section provides QC results in plain text, static plot, and interactive plot formats accessible by clicking on the corresponding hyperlinked texts, the iconized figures, and pointing hand, respectively. The Parallel Plot of QC values offers an integrated view of linked QC measures for a single sample or a group of samples (see also Fig. 4). The Expression Tables section provides links to raw read counts, a normalized RPKM table, and interactive display of gene expression levels (see also Fig. 6)

A screenshot of the entry webpage for the results of the test run is shown in Fig. 2. The page uses Noozle’s presentation template, which collates sections into a single neat web page with functionalities to expand or collapse individual or whole sections. In the “QC Metrics” section, both static images and interactive plots are provided for a variety of QC measures, including read mapping summaries, read counting statistics, SNP correlations among samples, number of expressed genes at various RPKM cutoffs, and correlations among gene expression profiles. All static QC plots can be enlarged into a new window by clicking on the iconized image, and the corresponding more dynamic and interactive plots are accessible by clicking the pointing hand icon. The interactive plots of QC measures offer many interactive features over static images, such as zooming in and zooming out. The raw data that was used to generate these figures can be accessed simply by clicking the corresponding hyperlinked text. The “Parallel Plot” and “Expression Table” sections in Fig. 2 are detailed later. Furthermore, all the result files and figures are directly accessible by expanding the “Raw Data Files” section shown at the bottom of Fig. 2. The entry webpage makes data navigation and visualization more convenient and intuitive, especially for experimental scientists.

#### SNP correlation to detect mishandled samples

**Fig. 3:**
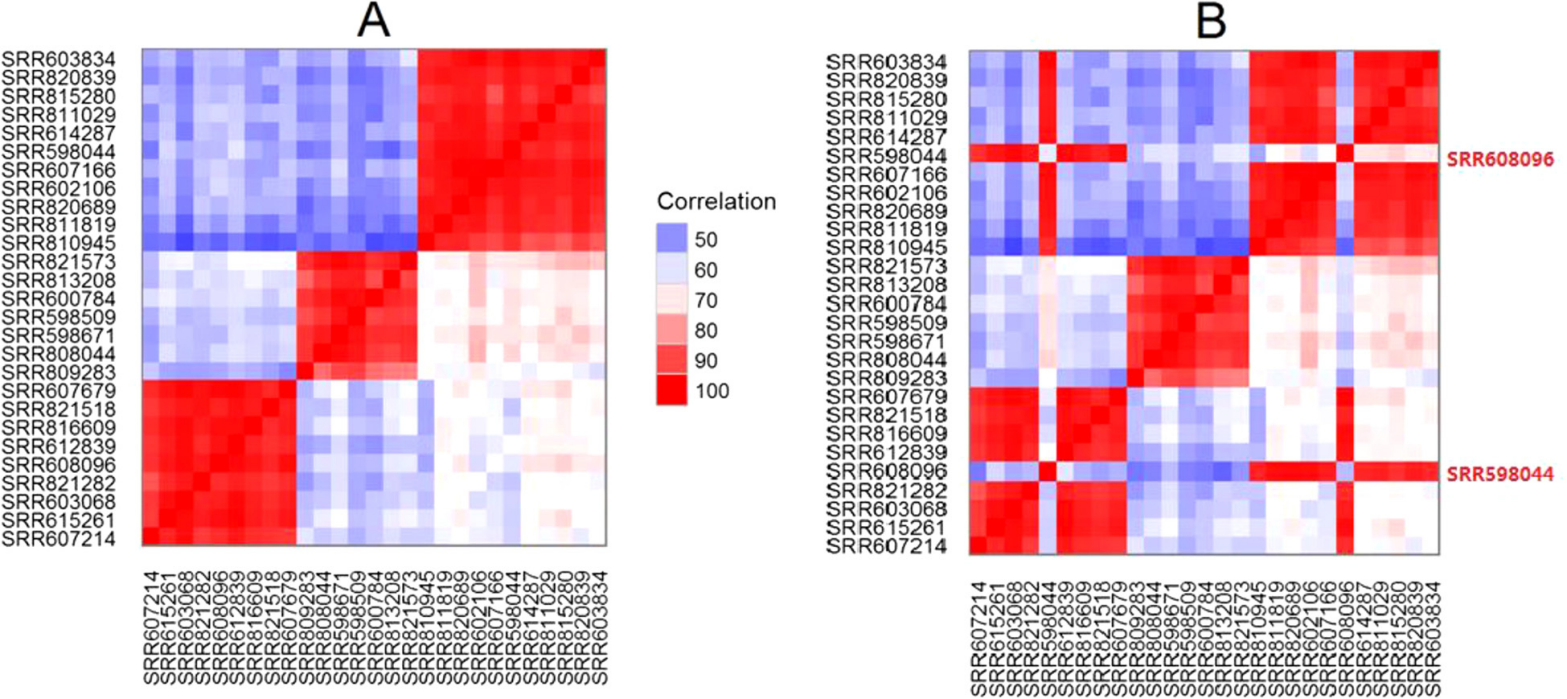
Representative SNP correlation plots to detect sample swapping. **a** Samples are nicely clustered by donors, as expected. **b** Clustering is disrupted after purposely swapping SRR598044 and SRR608096

SNP correlation plots help to verify whether samples are from the same subject or not. By definition, SNP concordance among samples from the same subject will be much higher than those samples from different subjects. In the first case, typical examples may be samples of different tissues from the same subject or longitudinal samples from the same subject. For simplicity, we selected samples from three donors to illustrate the usefulness of the SNP concordance plot (Fig. 3). As we expected, the SNP correlation plot in Fig. 3a is clustered by the donors. The corresponding correlation plot after the swap of SRR598044 and SRR608096 is shown in Fig. 3b where the correlation pattern indicates that the two samples are wrongly labeled. The true identifiers for the two swapped samples are indicated on the right of the plot. Sample swapping is typically very difficult to detect when it occurs. We have tried different methods to rectify mislabeled or swapped samples and found that a SNP correlation-based approach gave the best results (data not shown).

#### Integrated QC metrics for individual sample

**Fig. 4:**
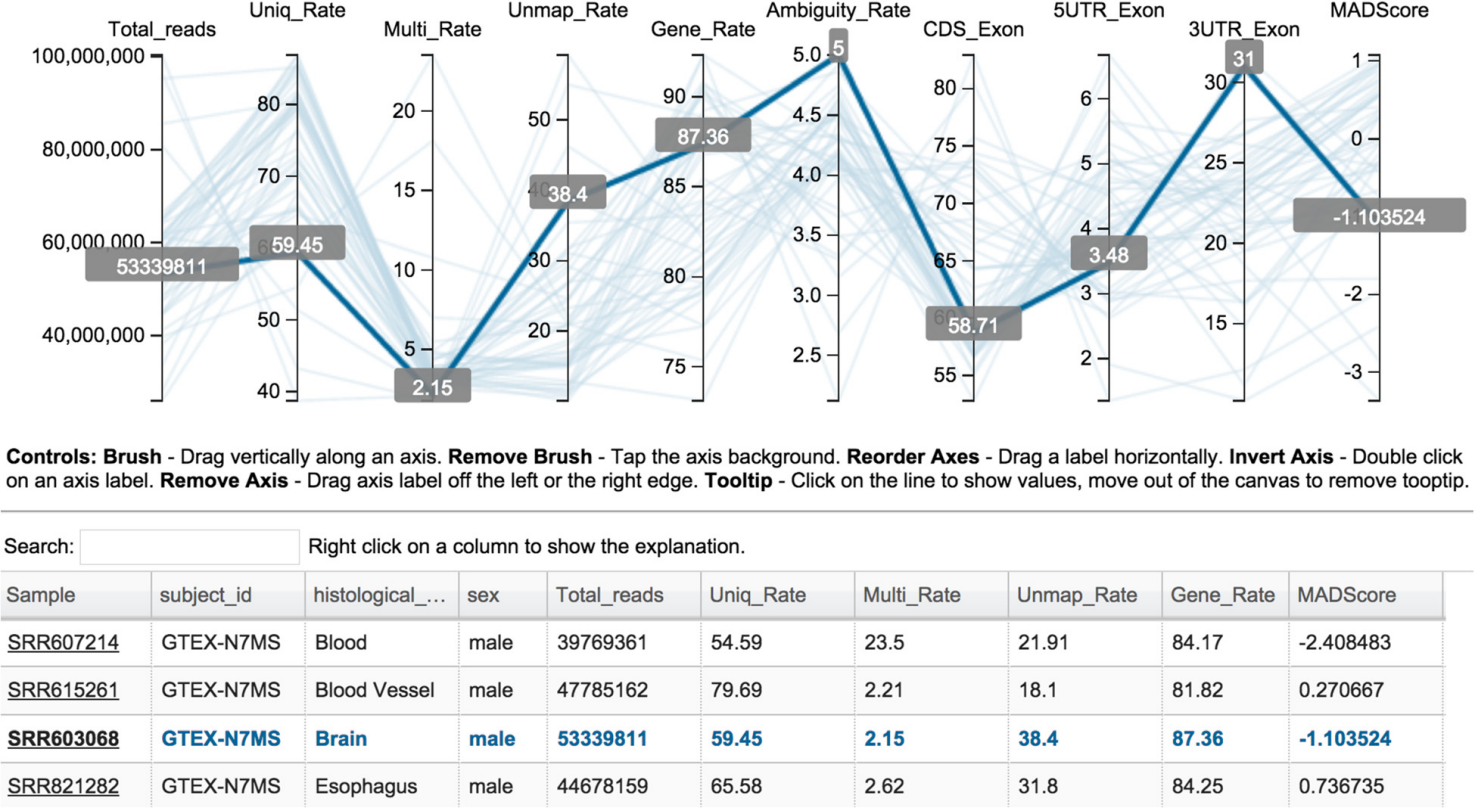
Parallel plot and table of multi-dimensional QC measures. Top panel displays one representative sample with each measure shown in a shaded tooltip. Bottom panel provides sample annotation and the full QC measures in a searchable table. Hovering the cursor over a sample in the table highlights the corresponding sample in the parallel plot. Parallel plots can be customized using the controls instructions below the plot

The parallel plot in Fig. 4 is a common way to visualize high-dimensional data and it is used widely in multivariate data analysis. We implemented the parallel plot to link all related QC measurements for all samples into one plot. Each axis within the plot represents a sample feature or a QC measurement. There are multiple ways users can control the look and feel of the plot, such as selecting a subset of samples to view, changing the order of the axes by drag-and-drop, and removing unwanted axes for a clearer view by dragging them off the plot to either side. The linked table is searchable, and for any selected sample in the table, its corresponding QC measures are highlighted simultaneously on the plot with tooltips showing the measurement values.

**Fig. 5:**
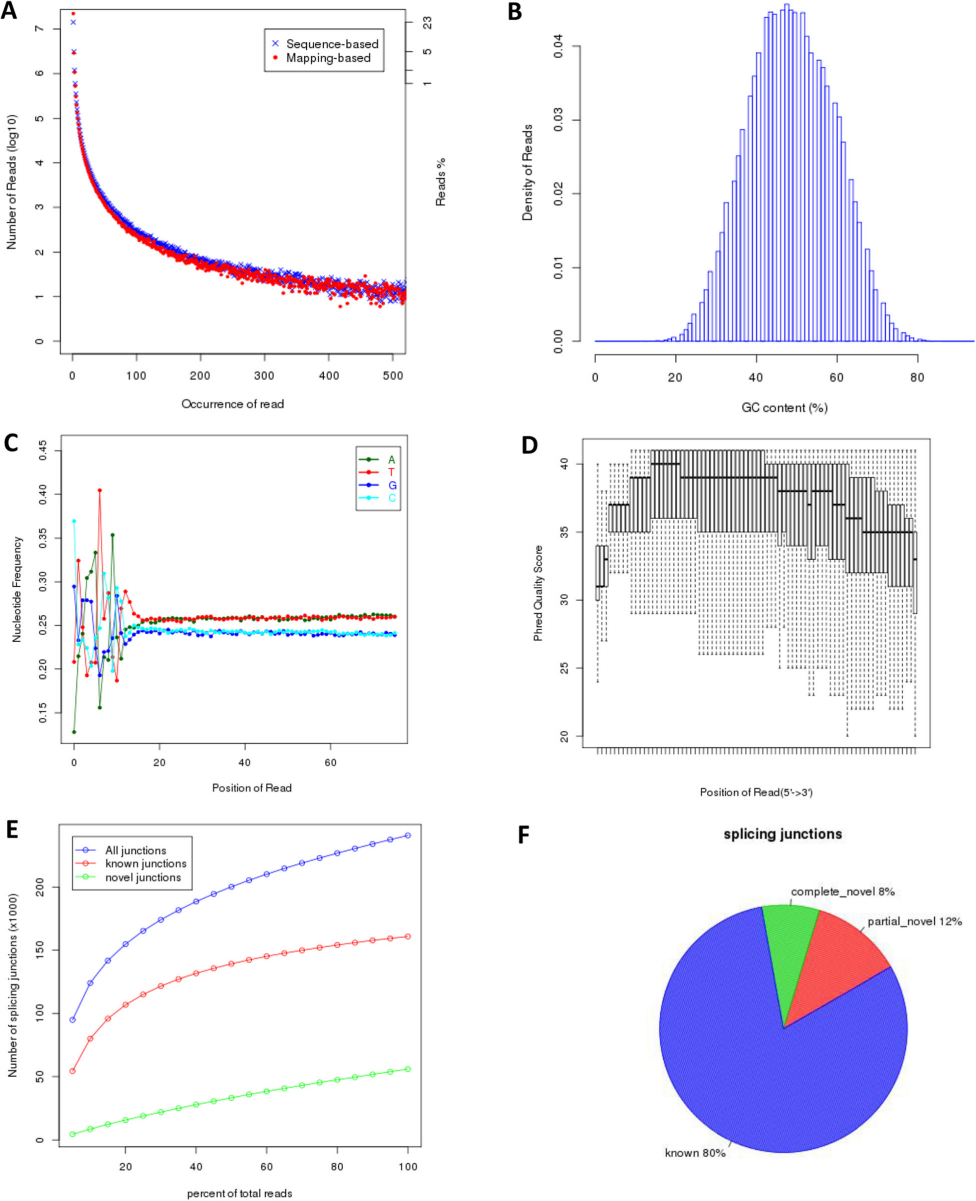
RNA-seq quality control metrics for the SRR603068 sample. **a** Duplication rates of the reads determined using a sequence-based and a mapping-based strategy. **b** Distribution of reads based on their percentage GC content. **c** Nucleotide composition bias of the reads. **d** Distribution of read quality scores. **e** Plot of junction saturation among the reads. **f** Characteristics of the splicing junction sites

MAD, an alternative and more robust measure of dispersion has been proposed to detect outliers [41]. We extended MAD to implement a correlation-based QC to detect potential outliers. The MADScore was calculated as described above, and is listed in the table in Fig. 4. To determine whether a potential outlier identified from the correlation-based QC is a true outlier, we recommend that the corresponding QC report is also checked. The comprehensive QC report for an individual sample can be accessed by clicking the corresponding sample identifier in the table in Fig. 4. For example, some representative RNA-seq QC metrics for SRR603068 (highlighted in Fig. 4) are shown in Fig. 5. The metrics correspond to reads duplication rate, distribution of reads versus percentages of GC content, nucleotide composition bias, distribution of read quality score, plot of junction saturation, and characteristics of the splicing junction sites.

Two strategies are used to determine the read duplication rate, as indicated in Fig. 5a. For the sequence-based strategy, reads with exactly the same sequence content are regarded as duplicated reads, whereas, for the mapping-based strategy, reads mapped to the same genomic location are regarded as duplicated reads. For spliced reads, reads mapped to the same starting position that splice the same way are regarded as duplicated reads. SRR603068 is a brain sample, and its nucleotide composition is biased towards A/T, as indicated in Fig. 5c. For RNA-seq data, we often want to know whether the sequencing depth is enough for the analyses, and the saturation plot shown in Fig. 5d is very valuable for this. For a well annotated organism, the number of expressed genes in a particular tissue is almost fixed so the number of splice junctions is also fixed. These numbers should be rediscovered from saturated RNA-seq data. The plot in Fig. 5d indicates that more reads should be sequenced for performing alternative splicing analyses. In Fig. 5f, all multiple splicing events spanning the same intron have been consolidated into one splicing junction, and a novel junction is considered as *complete_novel* if neither of the two splice sites can be annotated by a gene model. Otherwise, it is *partial_novel*, meaning that one of the splice site (5′SS or 3′SS) is new, while the other splice site is annotated (known). While the majority of junctions in Fig. 5f are annotated, over 20 % are either *complete_*novel or *partial_novel*.

#### Interactive visualization of gene expression profiles

**Fig. 6:**
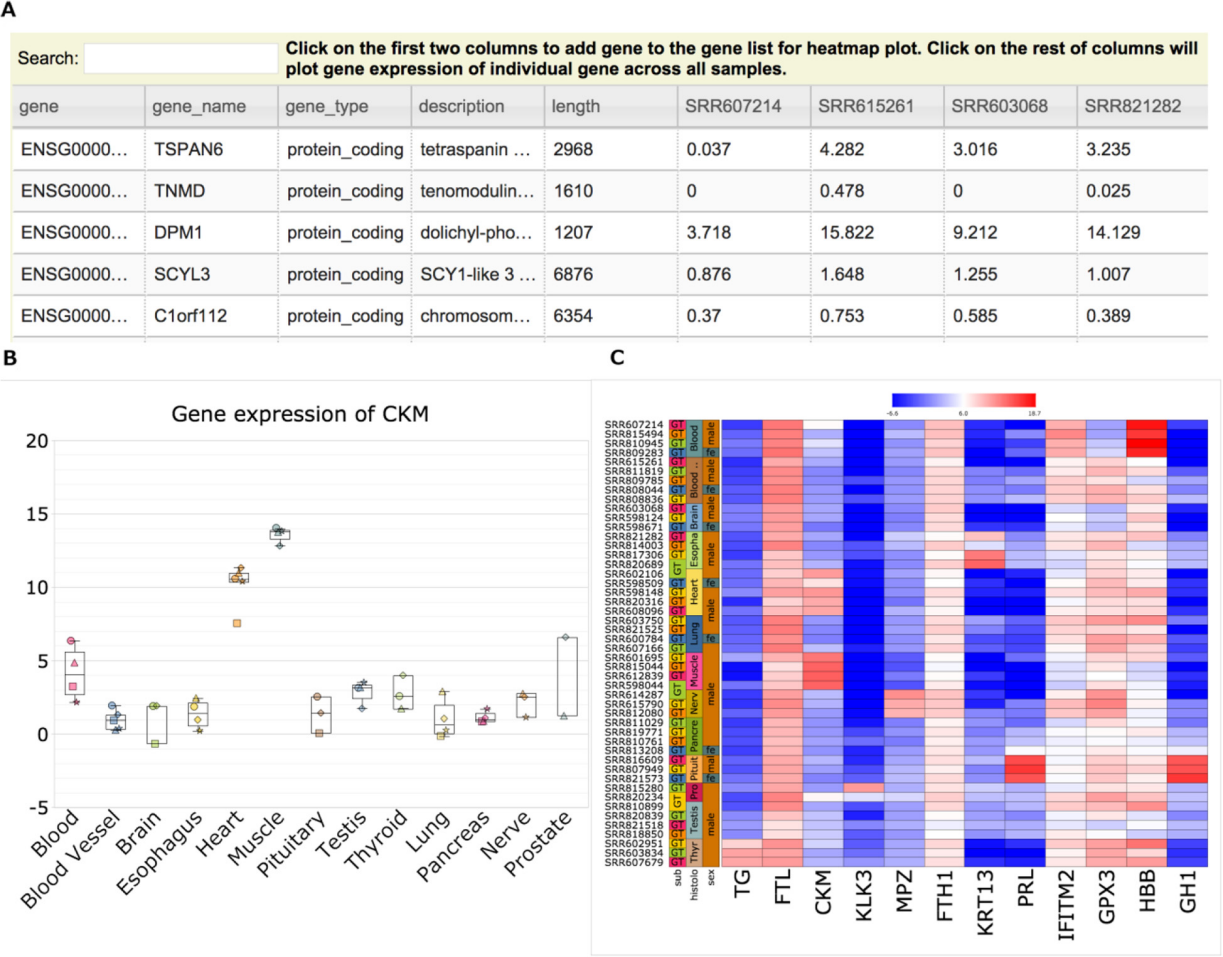
Interactive visualization of gene expression. **a** Gene expression levels of selected genes are displayed in a searchable table. **b** Boxplot view of the expression levels of CKM (creatine kinase, muscle). **c** Heat map view of gene expression levels of selected genes. Expression values can be grouped or split according to the sample annotations, such as tissue type. Each plot is highly customizable on the fly by right clicking on the plot and selecting relevant options from the dropdown menu

One of the most important objectives in many RNA-seq studies is to estimate gene expression levels under certain biological or disease conditions. With the help of the visualization tools shown in Fig. 6, differences in gene expression levels across samples under different conditions can be highlighted easily by a few mouse-clicks either in the boxplot (Fig. 6b) or heat map view (Fig. 6c). A keyword search box at the top of the table (Fig. 6a) provides an easy way to look at related genes such as kinases and interleukins. Gene expression profiles can be grouped and split on the fly according to the sample annotations, such as tissue type, visiting time, and treatment arms. Moreover, the look and feel of a plot, such as font size, color, plot type, and scales for x-axis and y-axis, can be customized by right clicking on the plot and selecting relevant options from the dropdown menu. An annotated heat map (Fig. 6c) is informative in comparing gene expression profiles across different conditions, and can help reveal the relationships between gene expression levels and corresponding biological conditions. Detailed instructions on how to use advanced visualization features of the interactive plot are described in the QuickRNAseq user guide that is bundled with the QuickRNASeq package.

### Scalability of QuickRNASeq

All samples can be processed in parallel in Step #1 of the QuickRNAseq pipeline (Fig. 1). In principle, there is no limitation to the number of RNA-seq samples, as long as enough storage is available. For easy data sharing, the web 2.0 visualization tools allow user to interact with the analyses results without the need of a web server and/or database. Therefore, in QuickRNAseq we pack all the data into JavaScript objects within a HTML document. For a RNA-seq project with 1000 samples, the number of gene expression data points can exceed 20 million, assuming that more than 20,000 genes are expressed. As a result, most browsers such as Internet Explorer, Safari, Firefox, and Chrome fail to load such huge datasets because they surpass the memory limit allocated to these web browsers. To solve this problem, we used pako [42], a web-based compression technique, to significantly reduce the number of objects to be created without compromising the end user experience.

### Limitations and running of QuickRNASeq

QuickRNASeq is presumed to be executed in a HPC environment, which can process multiple samples in parallel. The out-of-the-box QuickRNASeq pipeline has been fully tested in a HPC computing environment using the IBM Platform’s Load Sharing Facility (LSF) [43], a powerful workload management platform for demanding, distributed HPC environments. The IBM Platform’s LSF provides a comprehensive set of intelligent, policy-driven scheduling features that enable users to utilize all the computing infrastructure resources and ensure optimal application performance. In addition to LSF, many other notable job scheduling software are available [44]. For a cluster that uses a job scheduler other than LSF, *star-fc-qc.sh* (implementation of Step #1 in Fig. 1) needs to be customized accordingly. The only required change in the script is the way of job submission, and this command is dependent the job scheduling software. For researchers with no access to a HPC computing environment, we implemented *star-fc-qc.ws.sh*, a customized script that runs on a standard Linux workstation. Of course, analyzing large RNA-seq datasets on a single workstation is not typical and not recommended.

For gene quantifications, QuickRNAseq requires a complete genome sequence and well-annotated genes as inputs. The pipeline is not intended for the discovery of novel isoforms. QuickRNASeq is designed for use by bioinformaticians, experimental biologists, and geneticists in the fields of genome-scale analysis, functional genomics, and systems biology; however, downloading, installing, and running the QuickRNASeq pipeline in a Linux environment will require some basic computer-based expertise. A *README.txt* is provided along with the QuickRNASeq package, which explains step-by-step how to run QuickRNASeq. In addition, users can examine the configuration and sample annotation file under the *test_run* folder in the QuickRNASeq package. QuickRNASeq can be run without a sample annotation file, but it is strongly recommended that users provide meaningful annotations for all samples. A proper annotation file should be tab delimited, and QuickRNASeq requires that the first and second columns correspond to sample and subject identifiers, respectively. Sample names should start with a letter, and should not contain any white spaces.

In QuickRNASeq, we selected FeatureCounts, a union exon based approach, for gene quantification. According to our own most recent research [25], union exon based approach is discouraged. Unfortunately, there is still a long way to go for the switch from union exon based approach to transcript-based method in estimation of gene expression levels because of the inaccuracy of isoform quantification [25], especially for those isoforms with low expression, and gene-based annotation databases. Traditionally, functional enrichment analyses rely upon annotation databases such as Gene Ontology (GO) [45], Kyoto Encyclopedia of Genes and Genomes (KEGG) pathways [46] and other commercial knowledge systems. All such annotations have been recorded and centered on genes, not transcripts or isoforms. In practical RNA-seq data analyses, the switch from gene to isoform in quantification should ideally go with the switch in annotation hand by hand.

The current version of QuickRNASeq focuses on the automation of primary processing steps in RNA-seq data analyses, and these steps are in general biological question independent. We plan to expand QuickRNASeq to downstream analyses in the future, including differential analysis and pathway enrichment. Downstream analyses are usually driven by biological questions and experimental designs and thus different from project to project. How to automate such analyses in a user friendly manner remains a challenge for our practical implementation.

### QuickRNASeq versus QuickNGS

While this paper was in preparation, Wagle et al. [47] published QuickNGS, a new workflow system to analyze data from multiple next-generation sequencing (NGS) projects at a time. QuickNGS uses parallel computing resources, a comprehensive backend database, and the careful selection of previously published algorithmic approaches to build fully automated data analysis workflows. An overview of our comparison of the QuickRNASeq pipeline with the QuickNGS workflow is provided in Table 3. In summary, compared with QuickNGS, QuickRNASeq is more tailored to RNA-seq data. In QuickRNASeq, we developed scripts to perform RNA-seq-specific data integration and to generate integrated and interactive project reports in a fully automated manner. All the results from QuickRNASeq can be shared easily and further explored from a web browser on a personal computer even without internet access. Our pipeline QuickRNASeq provides a noticeable advancement of RNA-seq data analyses by incorporating a high degree of automation together with interactive visualizations.

**Table 3:** Comparison of QuickRNASeq with QuickNGS

	QuickNGS [41]	QuickRNAseq
Scope and application	Next-generation sequencing: WGS, RNA-seq, miRNA-seq, Chip-seq	RNA-seq only
Dependence	Requires external MySQL database and web server support	None
Purpose of web interface	Track the progress of data analysis and provide access to result files	Provide access to analyses results and interactive visualization
Visualization	Limited	Interactive, very rich and dynamic interface built upon web 2.0 technology
RNA-seq functionalities	Limited. Reduction of the hands-on time	“ONE-STOP” integrated report. Particularly implemented to support large-scale RNA-seq. High level of automation and efficiency

## Conclusions

By combing the best open source tool sets developed for RNA-seq data analyses and the most advanced web 2.0 technologies, we implemented the QuickRNASeq pipeline, which significantly reduces the efforts involved in primary RNA-seq data analyses and generates an integrated project report for data sharing and interactive visualization. The dynamic visualization features enable end users to explore and digest RNA-seq data analyses results intuitively and interactively, and to gain deep insights into RNA-seq datasets. The configuration file contains project, species, and software related parameters, and thus improves the reproducibly in RNA-seq data analyses. We have already applied QuickRNASeq to in-house large scale RNA-seq projects, and its current version is stable and mature for public release and adoption.

## Availability of software and supporting Information

**Project name:** QuickRNASeq pipeline

**Project home page:**http://quickrnaseq.sourceforge.net

**Operating system:** Linux

**Programming languages:** Bash scripting, Perl, R, JavaScript

**Dependencies:** R packages edgeR, reshape2 and ggplot2

**Other requirements:** None

**License:** GNU GPL version 3
